# Neonatal Hypoglycemia and Long-Term Pediatric Neurodevelopmental Outcomes: A Systematic Review

**DOI:** 10.7759/cureus.86183

**Published:** 2025-06-17

**Authors:** Rasha Fawzy Abdelmonem Mahrous, Sally Hassan Ali Hassanin, Raheeq Elssammani Elemam Elbashir, Hind Gasm Elseed, Sarra Elnour Ahmed Elnour, Nojoud Noureldayim Elsayid

**Affiliations:** 1 Neonatal Intensive Care Unit, King Abdulaziz Hospital, Jeddah, SAU; 2 Pediatric and Neonatal Intensive Care Unit, Al Ansari Specialized Hospital, Yanbu, SAU; 3 Pediatrics, University of Gezira, Wad Medani, SDN; 4 General Practice, GSM Medical Centre, Ajman, ARE; 5 Pediatrics, Abha Maternity and Children Hospital, Abha, SAU; 6 Pediatrics, Children Hospital, Ministry of Health, Riyadh, SAU

**Keywords:** child development, long-term outcomes, neonatal hypoglycemia, neurodevelopment, systematic review

## Abstract

Neonatal hypoglycemia is a common metabolic disturbance with potentially significant implications for neurodevelopment, yet the long-term consequences are not completely understood. This systematic review synthesises evidence from 13 studies to evaluate the association between neonatal hypoglycemia and neurodevelopmental outcomes in children, examining the roles of severity, timing, and clinical management. A comprehensive search across PubMed, Embase, Scopus, and Web of Science yielded 260 records, with 13 studies meeting inclusion criteria after rigorous screening. Methodological quality was assessed using the Newcastle-Ottawa Scale (NOS), revealing that seven studies had a low risk of bias, while six demonstrated a moderate risk. Findings indicate that severe hypoglycemia, particularly when early-onset or recurrent, is consistently associated with adverse outcomes, including motor dysfunction, cognitive delays, and executive function impairments. In contrast, milder hypoglycemia showed no consistent association with neurodevelopmental deficits when promptly treated. Heterogeneity in definitions and assessment methods across studies underscores the need for standardised criteria. The review highlights the importance of vigilant monitoring and targeted intervention for high-risk infants while suggesting that aggressive management of transient hypoglycemia may be unnecessary. Future research should prioritise longitudinal designs, consensus definitions, and exploration of protective factors to refine clinical guidelines and optimise neurodevelopmental outcomes.

## Introduction and background

Neonatal hypoglycemia is among the most common metabolic disturbances encountered in the immediate postnatal period, affecting both term and preterm infants [1]. Characterised by abnormally low blood glucose levels, its early identification and management remain critical due to the heightened metabolic demands of the developing neonatal brain [2]. While transient hypoglycemia is often considered benign when promptly corrected, growing evidence suggests that even short episodes of low glucose may have lasting implications for neurodevelopment [3].

Despite standardised thresholds and treatment protocols, substantial heterogeneity exists in clinical definitions, diagnostic timing, and intervention thresholds across institutions [4]. This inconsistency has complicated the establishment of clear associations between neonatal hypoglycemia and long-term neurodevelopmental outcomes such as cognitive function, executive processing, motor skills, and behavioral regulation [5]. Several observational and cohort studies have reported associations with adverse outcomes, including developmental delay, learning disabilities, and decreased school performance, whereas others suggest no significant long-term sequelae when hypoglycemia is effectively managed [6,7].

The primary objectives of this review are threefold: (1) to evaluate the association between neonatal hypoglycemia and long-term neurodevelopmental outcomes, (2) to assess whether this association varies by hypoglycemia severity, timing, or treatment, and (3) to identify critical gaps in the current evidence base to guide future research. Our findings have important implications for clinical practice, particularly in determining which infants may benefit from closer monitoring, earlier intervention, and targeted developmental support.

## Review

Methodology

This systematic review was conducted in accordance with the Preferred Reporting Items for Systematic Reviews and Meta-Analyses (PRISMA) 2020 guidelines [8].

Eligibility Criteria

The review included observational cohort studies (prospective and retrospective) and randomised controlled trials (RCTs) that examined the relationship between neonatal hypoglycemia and neurodevelopmental outcomes in children. Eligible studies had to meet the following criteria: (1) a defined population of neonates (term or preterm) with documented hypoglycemia, (2) a comparison group (euglycemic infants or different hypoglycemia thresholds), (3) assessment of neurodevelopmental outcomes (cognitive, motor, behavioral, or academic) at any age beyond infancy, and (4) publication in English in a peer-reviewed journal. Case reports, reviews, and studies without a control group were excluded.

Search Strategy

A comprehensive literature search was conducted in PubMed, Embase, Scopus, and Web of Science. The search strategy combined Medical Subject Headings (MeSH) and keywords related to neonatal hypoglycemia ("neonatal hypoglycemia," "transient hypoglycemia," "low blood glucose") and neurodevelopmental outcomes ("neurodevelopment," "cognitive impairment," "motor delay," "executive function"). The full search syntax was adapted for each database and is provided in the appendices section of this article (Appendix A).

Study Selection

After removing duplicates, two independent reviewers screened titles and abstracts for relevance. Full texts of potentially eligible studies were then assessed against the inclusion criteria. Conflicts were resolved through discussion or consultation with a third reviewer. The study selection process was documented in a PRISMA flow diagram, detailing the number of records identified, excluded, and included at each stage.

Data Extraction

A standardised data extraction form was developed and pilot-tested on three randomly selected studies to ensure consistency and comprehensiveness in data collection. The form captured key variables including study characteristics (author, year, country, study design, and sample size), population details, exposure assessment, and neurodevelopmental outcomes. To minimise bias and enhance reliability, two reviewers independently extracted data using this form, with any discrepancies between reviewers being resolved through discussion and consensus. This rigorous approach ensured the accurate and systematic collection of relevant data from all included studies while maintaining methodological consistency throughout the review process.

Risk of Bias Assessment

The methodological quality of included studies was assessed using the Newcastle-Ottawa Scale (NOS) [9] for cohort studies, a validated tool that evaluates three domains: selection (4 stars), comparability (2 stars), and outcome (3 stars), with a maximum score of 9 indicating the lowest risk of bias. Studies with a score of ≥7 were classified as high quality, those scoring between 5 and 6 as moderate quality, and those with a score of ≤4 were considered to have a high risk of bias.

Data Synthesis and Justification for No Meta-Analysis

Given the substantial clinical and methodological heterogeneity across studies, including varying definitions of hypoglycemia (e.g., different glucose thresholds, timing of exposure), diverse neurodevelopmental assessment tools, and differences in follow-up duration, meta-analysis was deemed inappropriate. Combining such heterogeneous data statistically would risk producing misleading pooled estimates. Instead, a narrative synthesis was conducted, categorising findings based on hypoglycemia severity, timing, and neurodevelopmental domains affected. This approach allowed for a nuanced interpretation of the evidence while acknowledging variations in study design and population characteristics.

Results

Study Selection

**Figure 1:**
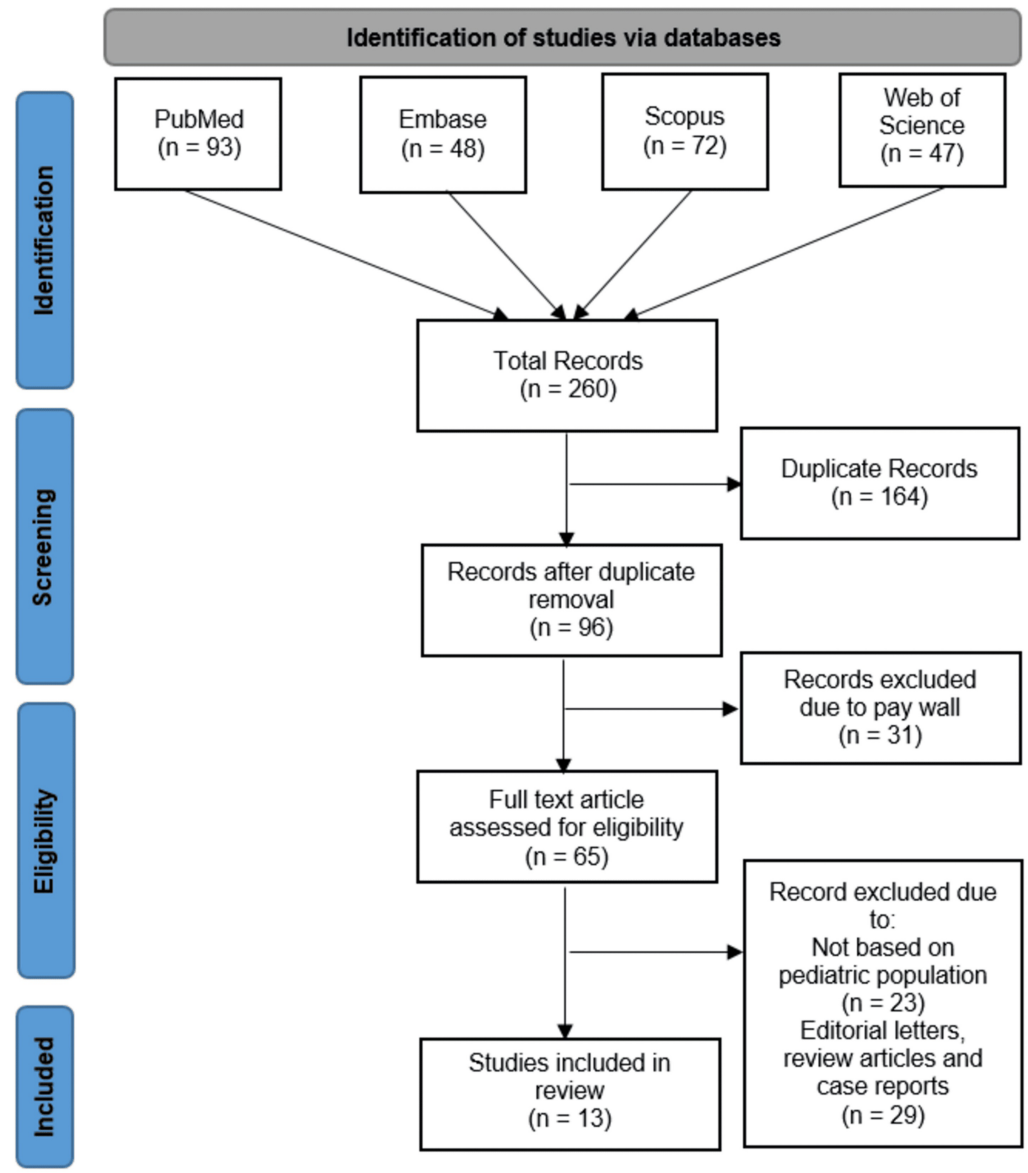
PRISMA 2020 flow diagram of literature search and screening process

The initial database search across Scopus (n=72), Embase (n=48), Web of Science (n=47), and PubMed (n=93) yielded 260 records, of which 164 duplicates were removed. After screening the remaining 96 records, 31 were excluded due to paywall restrictions, leaving 65 full-text articles assessed for eligibility. Of these, 23 were excluded for not focusing on pediatric populations, and 29 were removed as they comprised editorial letters, reviews, or case reports. Ultimately, 13 studies met the inclusion criteria and were incorporated into the systematic review (Figure 1).

Study Characteristics** **

We included 13 studies examining the association between neonatal hypoglycemia and long-term neurodevelopmental outcomes in children [10-22]. The studies varied in design, including prospective cohort studies [10,14-16,18-22], retrospective cohort studies [12], community-based stratified cohorts [11], and secondary analyses of randomised controlled trials [13]. Sample sizes ranged from 72 neonates [15] to 101,060 infants [17], with follow-up periods extending from 6 months to 10 years. The studies encompassed diverse populations, including term, preterm, and large-for-gestational-age (LGA) infants, and employed varying definitions of hypoglycemia, with glucose thresholds ranging from <1.7 mmol/L (<30 mg/dL) to <47 mg/dL (<2.6 mmol/L) (Table 1). 

**Table 1:** Summary of included studies characteristics. LGA: large for gestational age, NR: Not Reported, RCT: randomized controlled trial, IQ: intelligence quotient, OR: odds ratio, CI: confidence interval, ABC-2: movement assessment battery for children- second edition, Bayley III: Bayley scales of infant and toddler development-third edition, aRR: adjusted relative risk, USA: United States of America, mmol/L: millimoles per liter, mg/dL: milligrams per deciliter.

Author (year)	Country	Study design	Sample size	Gestational age (term/preterm)	Definition of Hypoglycemia	Timing of hypoglycemia assessment	Neurodevelopmental outcomes assessed	Age at follow-up	Key findings
Brand et al., [10] (2005)	Netherlands	Prospective observational	75	Term (LGA infants)	Plasma glucose <2.2 mmol/L at 1 hour, or <2.5 mmol/L thereafter	At 1, 3, and 5 hours after birth, continued if low	Denver developmental scale, non-verbal IQ test, child behavior checklist	4 years	No significant differences in developmental or behavior scores; lower reasoning IQ in one subscale but not consistent across definitions; no effect of treatment
Kerstjens et al., [11] (2012)	Netherlands	Community-based stratified cohort	832	Moderately preterm (32–35⁶⁄₇ weeks)	NR	Neonatal period (inferred from "neonatal morbidities")	Developmental delay via ages and stages questionnaire	43-49 months (approx. 3.5–4 years)	Hypoglycemia significantly associated with developmental delay (OR: 2.19; 95% CI: 1.08–4.46)
Kaiser et al., [12] (2015)	USA	Retrospective population-based cohort study	1395 newborn-student pairs (from 1943 eligible)	23–42 weeks’ gestation (includes both term and preterm)	Glucose level <35 mg/dL (primary), <40 mg/dL and <45 mg/dL (secondary)	Within the first 3 hours of life (early transient hypoglycemia)	Proficiency in 4th-grade literacy and mathematics achievement tests	10 years	Early transient hypoglycemia was associated with lower proficiency in literacy and math at 10 years of age
Goode et al., [13] (2016)	USA (national, multisite)	Secondary analysis of RCT longitudinal study	745 (with glucose data)	Preterm	Stratified into 4 glucose level groups (exact thresholds not specified)	Neonatal period (glucose levels recorded)	Cognitive, academic, behavioral assessments	3, 8, and 18 years	No significant differences in cognitive/academic skills; fewer problem behaviors at 18 in severe hypoglycemia group but clinically not meaningful
McKinlay et al., [14] (2015)	New Zealand	Prospective cohort Study	528	≥35 weeks (mostly term/preterm late)	Blood glucose <47 mg/dL (2.6 mmol/L)	Intermittent blood glucose for up to 7 days; continuous masked interstitial glucose monitoring	Bayley scales of infant development III; executive function; visual function	2 years	Neonatal hypoglycemia treated to maintain glucose ≥47 mg/dL was not associated with increased neurosensory impairment or processing difficulties at 2 years.
Mahajan et al., [15] (2017)	India	Prospective cohort study	72 hypoglycemic neonates (27 symptomatic, 45 asymptomatic) + 70 euglycemic controls	>32 weeks of gestation (both term and preterm included)	Plasma glucose < 50 mg/dL; also analyzed < 40 mg/dL threshold	First week of life (enrollment); neurodevelopment assessed at 6 and 12 months corrected age	Motor and mental development quotients; cerebral palsy diagnosis	6 months and 12 months corrected age	Hypoglycemia (both symptomatic and asymptomatic) is associated with significantly lower motor and mental scores compared to euglycemia. Worse outcomes with symptomatic hypoglycemia and glucose <40 mg/dL. 8% developed cerebral palsy in the hypoglycemic group.
McKinlay et al., [16] (2017)	New Zealand	Prospective cohort	614 neonates	Moderate to late preterm and term	Blood glucose <47 mg/dL; severe episode <36 mg/dL; recurrent ≥3 episodes; interstitial glucose <47 mg/dL for ≥10 minutes	Up to 7 days after birth (blood and interstitial glucose measured)	Cognitive function, executive function, visual function, and motor function	4.5 years	No increased risk of overall neurosensory impairment; increased risk of low executive function and visual motor function, especially with severe, recurrent, or clinically undetected hypoglycemia
Wickström et al., [17] (2018)	Sweden	Population-based cohort	101,060	Term (otherwise healthy infants)	Moderate neonatal hypoglycemia (exact glucose threshold not specified)	Early neonatal (<6 hours) and general neonatal period	Any neurological/neurodevelopmental outcome, developmental delay, motor delay, cognitive delay	2-6 years	Moderate neonatal hypoglycemia associated with increased risk of neurological and neurodevelopmental impairments; ORs up to ~3-fold higher for cognitive delay, especially with early hypoglycemia (<6h)
Qiao et al., [18] (2019)	China	Prospective cohort	195 infants enrolled; 157 followed with hypoglycemia; 144 control group	NR	Blood glucose level < 2.6 mM	Within 0.5 h after birth, subdivided into: <2 h, 2–24 h, >24 h after birth	Neurodevelopment evaluated by Gesell scoring method (focus on adaptability)	NR	Prolonged/repeated hypoglycemia (A2 and A3 groups) caused poor adaptability. Maternal insulin use and weight gain are associated with severe or persistent neonatal hypoglycemia.
Rasmussen et al., [19] (2020)	Denmark	Cohort follow-up with sibling controls	71 neonates with hypoglycemia, 32 healthy siblings	≥35 weeks (exclusion of <35 weeks)	Blood glucose <1.7 mmol/L (<30 mg/dL), treated to >2.5 mmol/L (>45 mg/dL)	Neonatal period (transient hypoglycemia recorded at the neonatal stage)	Cognitive function (Wechsler IV), motor function (Movement ABC-2), behavior (Child behavior checklist)	Mean 7.75 years (hypoglycemia), 9.17 years (siblings)	Lower fine motor scores in the hypoglycemia group within the normal range, especially boys; no difference in cognitive or behavioral outcomes
Edwards et al., [20] (2022)	New Zealand	Exploratory cohort analysis of RCT	1197	Late preterm and term	Blood glucose < 47 mg/dL; severe < 36 mg/dL; recurrent = ≥3 episodes	Neonatal period, screening and treatment to maintain glucose ≥47 mg/dL	Neurosensory impairment (blindness, deafness, cerebral palsy, developmental delay, executive function); Bayley III cognitive and motor scores	Corrected age 24 months	Hypoglycemia associated with increased risk of neurosensory impairment (aRR 1.28), especially severe episodes (aRR 1.68); no increased risk for recurrent episodes; lower Bayley-III cognitive and motor scores observed
Kennedy et al., [21] (2023)	New Zealand	Prospective cohort	101	≥36 weeks’ gestation (mostly term)	≥1 hypoglycemic episode with blood glucose <2.6 mmol/L or ≥10 min interstitial glucose <2.6 mmol/L	Neonatal period (exact timing not specified, but neonatal hypoglycemia episodes)	Executive function, academic achievement, emotional-behavioural regulation, visual memory, prosocial behaviour	9-10 years	Smaller caudate volume linked with greater emotional and behavioural difficulties and poorer prosocial behaviour. Neonatal hypoglycemia associated with smaller caudate volume but not with clinically relevant neurodevelopmental deficits.
Shah et al., [22] (2022)	New Zealand (Waikato hospital)	Prospective cohort study	614 recruited (480 assessed at follow-up)	Moderate to late preterm and term infants	Blood glucose <47 mg/dL (2.6 mmol/L) or interstitial sensor glucose <47 mg/dL for ≥10 minutes (episodes >20 min apart)	Up to 7 days after birth, continuous glucose monitoring with masked sensors	Primary: Educational achievement (reading comprehension, mathematics); Secondary: executive function, visual-motor function, psychosocial adaptation, general health	Mean 9.4 years	No significant difference in low educational achievement between hypoglycemia-exposed and unexposed groups; hypoglycemia-exposed children less likely rated below curriculum in reading by teachers.

*Neurodevelopmental Outcomes by Hypoglycemia Severity* 

The severity of neonatal hypoglycemia appeared to influence neurodevelopmental outcomes. Severe hypoglycemia (glucose <36 mg/dL or <2.0 mmol/L) was associated with adverse effects, including lower motor and mental development quotients at 6 and 12 months [15], increased risk of neurosensory impairment [20], and poorer executive and visual-motor function at 4.5 years [16]. Symptomatic hypoglycemia was linked to worse outcomes compared to asymptomatic cases, with an 8% incidence of cerebral palsy in the hypoglycemic group [15]. In contrast, milder hypoglycemia (glucose<47 mg/dL) was not consistently associated with neurodevelopmental deficits. For instance, McKinlay et al. [14] found no increased risk of neurosensory impairment at 2 years, and Shah et al. [22] reported no significant differences in educational achievement at mid-childhood. However, Wickström et al. observed a 3-fold higher risk of cognitive delay even with moderate hypoglycemia, particularly when occurring within the first 6 hours of life [17]. 

Timing of Hypoglycemia** **

The timing of hypoglycemia onset emerged as a critical factor. Early hypoglycemia (<6 hours postpartum) was associated with a higher risk of neurodevelopmental impairments, including cognitive and motor delays [17]. Similarly, Kaiser et al. [12] reported that transient hypoglycemia within the first 3 hours of life was linked to lower proficiency in literacy and mathematics at 10 years. Prolonged or recurrent hypoglycemia episodes also showed negative effects. Qiao et al. [18] found that hypoglycemia lasting >24 hours (A3 subgroup) resulted in poorer adaptability scores, while McKinlay et al. [16] noted that recurrent episodes (≥3) were associated with executive function deficits. 

Specific Neurodevelopmental Domains Affected 

The impact of neonatal hypoglycemia varied across neurodevelopmental domains. Cognitive outcomes were mixed; some studies reported no significant differences [10,13,19], while others found lower cognitive scores [20] or delays [17]. Motor function was more consistently affected, with lower fine motor scores [19] and increased cerebral palsy risk [15]. Executive function and visual-motor skills were particularly vulnerable, with impairments observed at 4.5 years [16] and smaller caudate volumes linked to emotional-behavioural difficulties at 9-10 years [21]. Behavioral outcomes were less frequently studied, though Goode et al. [13] noted fewer problem behaviors at 18 years in severe hypoglycemia cases, albeit without clinical significance. 

Heterogeneity in Findings** **

Notably, several studies reported no significant associations between neonatal hypoglycemia and long-term outcomes. For example, Brand et al. [10] found no overall negative effects in term LGA infants, and Shah et al. [22] observed no differences in academic performance. This heterogeneity may stem from variations in hypoglycemia definitions, treatment protocols, and follow-up durations. For instance, Edwards et al. [20] highlighted that aggressive treatment to maintain glucose ≥47 mg/dL mitigated some risks, underscoring the role of clinical management in shaping outcomes (Table 2). 

**Table 2:** Neurodevelopmental outcomes by hypoglycemia severity and timing. NR: not reported, OR: odds ratio, CI: confidence interval, IQ: intelligence quotient, ABC-2: movement assessment battery for children-second edition, mM: millimolar, mg/dL: milligrams per deciliter, mmol/L: millimoles per litre.

Author (year)	Hypoglycemia severity	Timing of onset	Glucose threshold used	Outcome domain affected	Direction of effect
Brand et al., [10] (2005)	Transient mild to severe	Within the first 1, 3, and 5 hours of birth	<2.2 mmol/L at 1 hour, <2.5 mmol/L afterwards	Reasoning IQ (subscale), Psychomotor development, behavior	No overall negative effect; slight decrease in reasoning IQ only
Kerstjens et al., [11] (2012)	NR	Neonatal period	NR	Developmental delay at preschool age	Increased risk (OR: 2.19; 95% CI: 1.08–4.46)
Kaiser et al., [12] (2015)	Transient (single low glucose)	Within the first 3 hours of life	<35, <40, and <45 mg/dL	Literacy and mathematics achievement	Decreased proficiency
Goode et al., [13] (2016)	More severe neonatal hypoglycemia (vs. mild/no hypoglycemia)	Neonatal period (preterm infants)	NR	Cognitive, academic, behavioral (age 3 to 18 years)	No significant difference in cognitive/academic, fewer behavioral problems at 18 in severe group (not clinically meaningful)
McKinlay et al., [14] (2015)	Neonatal hypoglycemia (blood glucose <47 mg/dL)	Neonatal period (up to 7 days)	47 mg per deciliter (2.6 mmol/L)	Neurosensory impairment, executive and visual function (processing difficulty)	No increased risk (risk ratio ~1, no adverse effect)
Mahajan et al., [15] (2017)	Symptomatic and asymptomatic	Neonatal period (first week of life)	<50 mg/dL (hypoglycemia), with special focus < 40 mg/dL	Neurodevelopmental outcomes: motor development quotient, mental development quotient, cerebral palsy incidence	Adverse effect (lower motor and mental development quotients; increased cerebral palsy in hypoglycemic infants)
McKinlay et al., [16] (2017)	Any hypoglycemia, severe (<36 mg/dL), recurrent (≥3 episodes), clinically undetected (interstitial episodes)	Neonatal period (birth to 7 days)	<47 mg/dL (blood), <36 mg/dL (severe), <47 mg/dL (interstitial for ≥10 min)	Executive function, Visual motor function, Neurosensory impairment (not increased)	Increased risk for executive and visual motor function impairment; no increased risk for combined neurosensory impairment
Wickström et al., [17] (2018)	Moderate neonatal hypoglycemia	Early neonatal (<6 hours) and overall neonatal period	NR	Any neurological or neurodevelopmental outcome; developmental delay (any, motor, cognitive)	Increased risk (OR >1 for all outcomes)
Qiao et al., [18] (2019)	Neonatal hypoglycemia overall (subgroups A1, A2, A3 with increasing severity)	<2h (A1), 2-24h (A2), >24h (A3) after birth	<2.6 mM	Neurodevelopment (adaptability)	Negative effect: poorer adaptability in A2 and A3 compared to controls
Rasmussen et al., [19] (2020)	Transient neonatal hypoglycemia	Neonatal period	<1.7 mmol/L (<30 mg/dL) for diagnosis; treated with >2.5 mmol/L (>45 mg/dL)	Fine motor function (Movement ABC-2 test), Cognitive function (Wechsler IV), behavior (child behavior checklist)	Lower fine motor scores within normal range, no significant changes in cognitive function or behavior; effect stronger in boys
Edwards et al., [20] (2022)	Any hypoglycemia, severe (<36 mg/dL), recurrent (≥3 episodes)	Neonatal period (first hour after birth)	<47 mg/dL (any), <36 mg/dL (severe), ≥3 episodes <47 mg/dL (recurrent)	Neurosensory impairment, cognitive and motor development	Increased risk for neurosensory impairment (especially severe), lower cognitive and motor scores; no significant risk increase for recurrent episodes
Kennedy et al., [21] (2023)	At least one hypoglycaemic episode (blood glucose <2.6 mmol/L or ≥10 min interstitial glucose <2.6 mmol/L)	Neonatal period (birth, within first days)	Blood glucose <2.6 mmol/L or interstitial glucose < 2.6 mmol/L for at least 10 minutes	Emotional-behavioural difficulties, prosocial behaviour, visual memory, executive function, academic achievement	Smaller caudate volume associated with greater emotional-behavioural difficulties and poorer prosocial behaviour; caudate volume positively associated with visual memory only in non-hypoglycaemic children; no relation to executive function or academic achievement
Shah et al., [22] (2022)	Moderate to mild hypoglycemia (blood glucose <47 mg/dL)	Neonatal period (first 7 days after birth)	Blood glucose <47 mg/dL (2.6 mmol/L)	Educational performance (reading comprehension, mathematics), executive function, visual-motor function, psychosocial adaptation, general health	No significant association with lower educational achievement; slight decreased risk for poor reading ratings by teachers

Risk of Bias Findings

The risk of bias assessment revealed that seven studies demonstrated particularly strong methodology with scores of 9/9, including Kerstjens et al. [11], Goode et al. [13], McKinlay et al. [14, 16], Wickström et al. [17], Rasmussen et al. [19], Edwards et al. [20], and Shah et al. [22]. These studies featured well-defined cohorts, appropriate control for confounding variables, and validated outcome measures. Six studies received moderate scores of 6-8/9 due to limitations such as incomplete adjustment for confounders or smaller sample sizes, including Brand et al. [10], Kaiser et al. [12], Mahajan et al. [15], Qiao et al. [18], and Kennedy et al. [21]. Notably, the study by Mahajan et al. [15] had a relatively small sample size (n=72), while Kaiser et al. [12] used a retrospective design that may have introduced selection bias. Despite these variations, the overall quality of evidence was robust, with most studies demonstrating low risk of bias, supporting the reliability of the systematic review's conclusions. The observed heterogeneity in hypoglycemia definitions across studies, with thresholds ranging from <1.7 mmol/L to <47 mg/dL, suggests the need for standardised criteria in future research (Table 3).

**Table 3:** Risk of bias findings using NOS tool.

Author (Year)	Selection (max 4)	Comparability (max 2)	Outcome (max 3)	Total score (max 9)	Risk of bias
Brand et al., [10] (2005)	3	1	2	6	Moderate
Kerstjens et al., [11] (2012)	4	2	3	9	Low
Kaiser et al., [12] (2015)	3	1	2	6	Moderate
Goode et al., [13] (2016)	4	2	3	9	Low
McKinlay et al., [14] (2015)	4	2	3	9	Low
Mahajan et al., [15] (2017)	3	1	2	6	Moderate
McKinlay et al., [16] (2017)	4	2	3	9	Low
Wickström et al., [17] (2018)	4	2	3	9	Low
Qiao et al., [18] (2019)	3	1	2	6	Moderate
Rasmussen et al., [19] (2020)	4	2	3	9	Low
Edwards et al., [20] (2022)	4	2	3	9	Low
Kennedy et al., [21] (2023)	4	2	2	8	Low
Shah et al., [22] (2022)	4	2	3	9	Low

Discussion

Our analysis of 13 studies reveals that while severe, early-onset, or prolonged hypoglycemia appears consistently associated with adverse neurodevelopmental sequelae, the evidence for milder or promptly treated hypoglycemia remains inconclusive. This dichotomy suggests that not all neonatal hypoglycemia carries equal risk, and that timing, severity, and clinical management may be critical moderators of outcomes. The most robust associations emerged for severe hypoglycemia (glucose <36 mg/dL or <2.0 mmol/L), which was linked to motor dysfunction, cognitive deficits, and in some cases, cerebral palsy [15,20]. These findings align with earlier work by Lucas et al. [23] that first established the potential for severe neonatal hypoglycemia to cause permanent neurological damage, particularly when prolonged or recurrent.

The temporal aspects of hypoglycemia emerged as particularly salient in our review. Early hypoglycemia occurring within the first 6 hours of life demonstrated stronger associations with adverse outcomes compared to later-onset episodes [12,17]. This timing effect may reflect the particular vulnerability of the neonatal brain during the immediate transitional period after birth, when cerebral glucose utilisation patterns are undergoing dramatic changes [24]. The finding that hypoglycemia during this critical window predicts poorer literacy and mathematics performance at age 10 years [12] suggests that early metabolic instability may disrupt fundamental neurodevelopmental processes that later manifest as academic difficulties. This temporal sensitivity also helps explain why some studies of later-onset or briefly treated hypoglycemia failed to find significant associations [14,22].

An important nuance in our findings concerns the differential impact across neurodevelopmental domains. Motor function appears particularly vulnerable to neonatal hypoglycemia, with multiple studies reporting impaired fine motor skills [19], visual-motor integration difficulties [16], and, in severe cases, cerebral palsy [15]. This pattern mirrors neuropathological studies showing selective vulnerability of motor pathways to hypoglycemic injury [25]. Cognitive outcomes were more variable, with some studies showing clear deficits [17,20] while others found no significant differences [10,13]. This inconsistency may reflect differences in the cognitive measures used, with more sensitive, domain-specific assessments (e.g., executive function) more likely to detect subtle deficits than global IQ measures [26].

The role of clinical management in modifying outcomes emerged as a critical consideration. Studies where hypoglycemia was aggressively treated to maintain glucose ≥47 mg/dL [14,16,20] generally found better outcomes than studies with less stringent treatment protocols. This suggests that current approaches to monitoring and treating neonatal hypoglycemia may be preventing some, though not all, of the potential neurodevelopmental consequences. However, the optimal threshold for intervention remains uncertain, as even carefully managed hypoglycemia was associated with subtle executive function difficulties in some studies [16,21]. These findings extend previous work by Tin [27], suggesting that while severe hypoglycemia clearly requires treatment, the risks of milder hypoglycemia may depend on additional factors such as duration and underlying neonatal vulnerabilities.

Population differences also appear important in interpreting these findings. The studies in our review included diverse populations from healthy term infants [10] to high-risk preterm neonates [13], and the impact of hypoglycemia varied accordingly. Preterm infants, who already face multiple neurodevelopmental risks, showed particular vulnerability to additional hypoglycemic insults [11,13]. This aligns with the concept of "double jeopardy" in neonatal neurology, where multiple risk factors interact to amplify neurodevelopmental risk [28]. Conversely, in otherwise healthy term infants, the neurodevelopmental impact of hypoglycemia appeared more modest [10,22], suggesting greater resilience or compensatory capacity in this population.

The neurobiological mechanisms underlying these associations warrant consideration. The finding that neonatal hypoglycemia is associated with reduced caudate volume and related emotional-behavioral difficulties at school age provides important clues about potential neural substrates [21]. The caudate nucleus, part of the basal ganglia, is rich in glucose transporters and particularly metabolically active during early development [29]. Its vulnerability to hypoglycemic injury may help explain some of the executive function and behavioral regulation difficulties observed in follow-up studies. Similarly, the visual-motor integration problems reported by McKinlay et al. may reflect hypoglycemia's impact on posterior cortical regions known to be sensitive to metabolic stress [16].

Our review highlights several important gaps in the current evidence base. First, there remains striking heterogeneity in how neonatal hypoglycemia is defined and measured across studies, ranging from single glucose measurements to continuous monitoring protocols. This variability makes comparisons across studies challenging and underscores the need for consensus definitions, as called for by the Pediatric Endocrine Society [30]. Second, few studies have examined potential protective factors that might mitigate hypoglycemia's effects, such as breastfeeding patterns or early developmental enrichment. Third, most studies have focused on early and middle childhood outcomes, with limited data on adolescent or adult functioning. This is particularly important given that some neurodevelopmental effects may not become apparent until higher cognitive demands emerge later in development.

The clinical implications of these findings are significant but nuanced. While our review supports vigilance for severe or prolonged neonatal hypoglycemia, it also suggests that the neurodevelopmental risks of milder, transient hypoglycemia may be overstated in some clinical contexts. This aligns with recent shifts toward more targeted approaches to neonatal glucose management [31]. However, the identification of specific high-risk scenarios, particularly early-onset, severe, or recurrent hypoglycemia, should inform clinical monitoring protocols and early intervention strategies. The finding that hypoglycemia's effects may be domain-specific (affecting motor and executive functions more than global cognition) suggests that follow-up assessments should include targeted evaluation of these vulnerable domains.

Limitations

Several limitations of this review must be acknowledged. First, the included studies varied considerably in their methodologies, populations, and definitions of hypoglycemia, limiting direct comparability. Second, despite our comprehensive search, we cannot exclude the possibility of publication bias favoring studies with positive findings. Third, most studies were observational in design, precluding definitive causal conclusions about the relationship between hypoglycemia and outcomes. Fourth, the review was limited to English-language publications, potentially omitting relevant studies published in other languages. Finally, the long-term significance of some of the more subtle neurodevelopmental differences reported remains uncertain, particularly in the absence of adult outcome data.

## Conclusions

Neonatal hypoglycemia, particularly when severe, early-onset, or prolonged, is associated with adverse neurodevelopmental outcomes across multiple domains. However, the risks appear moderated by factors including clinical management, population characteristics, and hypoglycemia severity and timing. These findings support current clinical guidelines emphasising prompt identification and treatment of significant neonatal hypoglycemia while suggesting that universal screening and aggressive management of mild, transient hypoglycemia may not be warranted. Future research should focus on establishing consensus definitions, identifying biomarkers of brain injury risk, and developing targeted intervention strategies for infants at the highest neurodevelopmental risk. Clinicians should remain vigilant for hypoglycemia in high-risk neonates while adopting a balanced approach that considers both the potential risks of hypoglycemia and the costs of over-treatment.

## Appendices

Appendix A 

**Table 4:** Search strategy for databases.

Database	Search string
PubMed	("neonatal hypoglycemia"[MeSH Terms] OR "neonatal hypoglycemia"[Title/Abstract] OR "newborn hypoglycemia"[Title/Abstract] OR "infant hypoglycemia"[Title/Abstract]) AND ("neurodevelopmental outcomes"[Title/Abstract] OR "neurological development"[Title/Abstract] OR "cognitive development"[Title/Abstract] OR "neurodevelopment"[MeSH Terms] OR "child development"[MeSH Terms]) AND ("long-term effects"[Title/Abstract] OR "follow-up studies"[MeSH Terms] OR "longitudinal study"[Title/Abstract] OR "developmental outcomes"[Title/Abstract])
Embase	'neonatal hypoglycemia'/exp OR 'neonatal hypoglycemia':ti,ab OR 'newborn hypoglycemia':ti,ab OR 'infant hypoglycemia':ti,ab AND ('neurodevelopment'/exp OR 'neurodevelopmental outcome':ti,ab OR 'cognitive development':ti,ab OR 'neurological development':ti,ab) AND ('long term outcome':ti,ab OR 'follow up study'/exp OR 'longitudinal study':ti,ab OR 'developmental delay':ti,ab)
Scopus	TITLE-ABS-KEY("neonatal hypoglycemia" OR "newborn hypoglycemia" OR "infant hypoglycemia") AND TITLE-ABS-KEY("neurodevelopmental outcome" OR "neurological development" OR "cognitive development" OR "child development" OR "neurodevelopment") AND TITLE-ABS-KEY("long-term outcome" OR "follow-up" OR "developmental delay" OR "longitudinal study")
Web of Science	TS=("neonatal hypoglycemia" OR "newborn hypoglycemia" OR "infant hypoglycemia") AND TS=("neurodevelopmental outcome" OR "neurological development" OR "cognitive development" OR "child development" OR "neurodevelopment") AND TS=("long-term effects" OR "follow-up studies" OR "longitudinal study" OR "developmental outcomes")
